# Percutaneous kidney biopsies in children: a 24-year review in a tertiary center in northern Portugal

**DOI:** 10.1590/2175-8239-JBN-2023-0143en

**Published:** 2024-04-08

**Authors:** Patrícia Sousa, Catarina Brás, Catarina Menezes, Ramon Vizcaino, Teresa Costa, Maria Sameiro Faria, Conceição Mota

**Affiliations:** 1 Hospital Senhora da Oliveira, Serviço de Pediatria, Guimarães, Portugal.; 2 Hospital Professor Doutor Fernando Fonseca, Serviço de Nefrologia, Lisboa, Portugal.; 3 Centro Hospitalar Universitário de Santo António, Centro Materno-Infantil do Norte, Serviço de Pediatria, Porto, Portugal.; 4 Centro Hospitalar Universitário de Santo António, Serviço de Anatomia Patológica, Porto, Portugal.; 5 Centro Hospitalar Universitário de Santo António, Centro Materno-Infantil do Norte, Unidade de Nefrologia Pediátrica, Serviço de Pediatria, Porto, Portugal.; 6 Unidade de Ciências Biomoleculares Aplicadas, Department of Biological Science, Porto, Portugal.

**Keywords:** Biopsy, Kidney, Pediatrics

## Abstract

**Introduction:**

Percutaneous kidney biopsy (KB) is crucial to the diagnosis and management of several renal pathologies. National data on native KB in pediatric patients are scarce. We aimed to review the demographic and clinical characteristics and histopathological patterns in children who underwent native percutaneous KB over 24 years.

**Methods:**

Retrospective observational study of patients undergoing native percutaneous KB in a pediatric nephrology unit between 1998 and 2021, comparing 3 periods: period 1 (1998–2005), period 2 (2006–2013), and period 3 (2014–2021).

**Results:**

We found that 228 KB were performed, 78 (34.2%) in period 1, 91 (39.9%) in period 2, and 59 (25.9%) in period 3. The median age at KB was 11 (7–14) years. The main indications for KB were nephrotic syndrome (NS) (42.9%), hematuria and/or non-nephrotic proteinuria (35.5%), and acute kidney injury (13.2%). Primary glomerulopathies were more frequent (67.1%), particularly minimal change disease (MCD) (25.4%), IgA nephropathy (12.7%), and mesangioproliferative glomerulonephritis (GN) (8.8%). Of the secondary glomerulopathies, lupus nephritis (LN) was the most prevalent (11.8%). In group 1, hematuria and/or non-nephrotic proteinuria were the main reasons for KB, as opposed to NS in groups 2 and 3 (p < 0.01). LN showed an increasing trend (period 1–3: 2.6%–5.3%) and focal segmental glomerular sclerosis (FSGS) showed a slight decreasing trend (period 1–3: 3.1%–1.8%), without statistical significance.

**Conclusions:**

The main indication for KB was NS, which increased over time, justifying the finding of MCD as main histological diagnosis. LN showed an increase in incidence over time, while FSGS cases did not increase.

## Introduction

Percutaneous kidney biopsy (KB) plays an important role in the diagnosis of kidney diseases, providing histopathological data to complement clinical assessment and assisting adequate diagnosis, treatment, and prognosis^1^. Kidney biopsy has proven to be a safe procedure, as severe complications following the procedure are rare, the most common of which being bleeding: perirenal hematoma (12.4%) and macroscopic hematuria (2.6%)^2–4^. Microscopic hematuria is found in up to 3.5% of patients. Fewer than 1% of patients require erythrocyte transfusion^5^.

Several studies show differences in the epidemiology of renal disease between adult and pediatric populations, as well as geographic variation^2,6^. Data regarding the general population are extensively available, whereas pediatric international and national records are more recent and scarce^7^. Therefore, reports of the epidemiology and histopathology of chronic kidney disease in children are crucial to guide our approach.

Glomerulonephritis represents a heavy burden in chronic pediatric kidney disease, second only to congenital abnormalities of the kidney and urinary tract^1^.

Studies conducted in different populations have pointed to minimal change disease (MCD), immunoglobulin A nephritis (IgAN), and immunoglobulin A vasculitis-nephritis (IgAVN) as the most common histopathological findings. The most frequent indication for KB in children appears to be nephrotic syndrome (NS)^1^ and or proteinuria^8^, although some studies report hematuria as the most common^9^.

In this study, we aimed to report the clinical indications and histopathological findings of percutaneous native KB in children in a tertiary pediatric center in northern Portugal, as well as analyze the evolution over a period of twenty-four years.

## Methods

This retrospective study included all patients submitted to a first percutaneous native KB in a tertiary pediatric nephrology center in northern Portugal from January 1st 1998 to December 31st 2021. Repeated biopsies in the same patient and biopsies performed on transplanted kidneys were excluded. All KB were performed with ultrasound guidance, by pediatric nephrologists up to 2012 and by interventional radiologists thereafter, due to organizational reform. There were no other significant changes in biopsy method during this time period. Informed written consent was obtained for all procedures.

Digital and on-paper clinical records of the included patients were reviewed to retrieve data regarding sex, age at time of diagnosis, age at time of biopsy, indication for percutaenous KB, presence and degree of hematuria, presence and degree of proteinuria, number of glomeruli obtained, immunofluorescence staining, electronic microscopy, and histological diagnosis. Indications for percutaneous KB were categorized as NS, asymptomatic urinary abnormalities (including non-nephrotic proteinuria, hematuria or both), acute renal failure (AKI), and chronic kidney failure (CKD).

Samples were examined by experienced pathologists and were considered valid if there were at least 7 glomeruli or otherwise if a diagnosis was made, according to institutional protocol and literature^10^.

Immunofluorescence staining using polyclonal antisera against human IgG, IgM, IgA, C3, C4, C1q, and albumin was performed.

Renal diseases were divided into four groups: 1) primary glomerulonephritis (GN); 2) secondary GN; 3) tubulointerstitial diseases, and 4) other diseases.

Primary GN included: crescent glomerulonephritis (CreGN), C1q nephropathy (C1qN), C3 glomerulopathy (C3G), focal segmental glomerulosclerosis (FSGS), idiopathic membranous nephritis, IgAN, immunoglobulin M nephropathy (IgMN), membranoproliferative glomerulonephritis (MPGN), mesangial proliferative glomerulonephritis (MsPGN), MCD, and thin basal membrane nephropathy (TMBN). Secondary GN included acute post-infectious glomerulonephritis (APiGN), IgAVN, and lupus nephritis (LN). Tubulointerstitial diseases included acute and chronic tubulointerstitial nephritis. Other diagnoses comprise thrombotic microangiopathy (TM), Alport syndrome (AS), congenital nephrotic syndrome (CNS).

The sample was divided in three time periods, according to time of biopsy: period 1 (1998–2005), period 2 (2006–2013), and period 3 (2014–2021).

For categorical variables, data are presented as frequencies and percentages. Continuous variables had a non-parametric distribution and are reported as medians and interquartile ranges. Groups were compared according to the Mann-Whitney U test. A *p*-value <0.05 was considered statistically significant. Statistical analysis was performed using the IBM SPSS Statistics software version 28.0.1.0 (SPSS Inc., Chicago, IL, USA).

This study was approved by the institution’s Ethics Committee and individual informed written consent was deemed unnecessary for this retrospective study.

## Results

**Figure 1:**
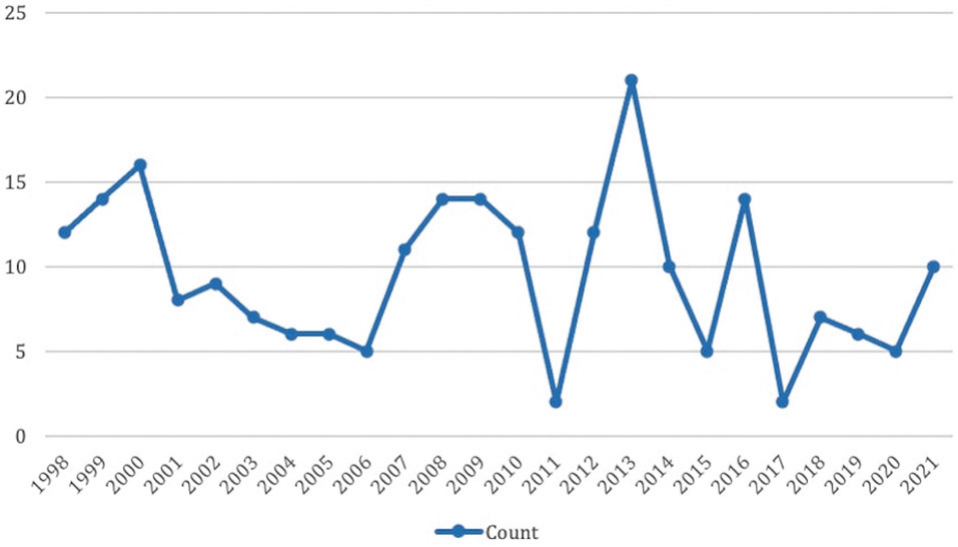
Evolution of percutaneous kidney biopsies performed from 1998-2021. Points represent the count of biopsies per year.

Overall, 228 native KB were performed, 78 (34.2%%) in period 1, 91 (39.9%) in period 2, and 59 (25.9%) in period 3. Distribution per year is represented in Figure 1.

Demographic characteristics are represented in Table 1. In our sample, 50.4% of patients were male (n = 115) and 49.6% were female (n = 113). Sex distribution was similar over the three time periods. Median age at time of biopsy was 11 (7–14), with no significant difference among time periods.

**Table 1:** Demographic characteristics

		Period 1	Period 2	Period 3	Total
Specimens		n = 78 (34.2%)	n = 91 (39.9%)	n = 59 (25.9%)	n = 228
Sex	Male	n = 37 (47.4%)	n = 49 (53.8%)	n = 29 (49.2%)	n = 115 (50.4%)
	Female	n = 41 (52.6%)	n = 42 (46.2%)	n = 30 (50.8%)	n = 113 (49.6%)
Age (years)		9 (6–13)	12 (8–14)	11 (7–14)	11 (7–14)

Numbers represent count (percentages).

**Figure 2:**
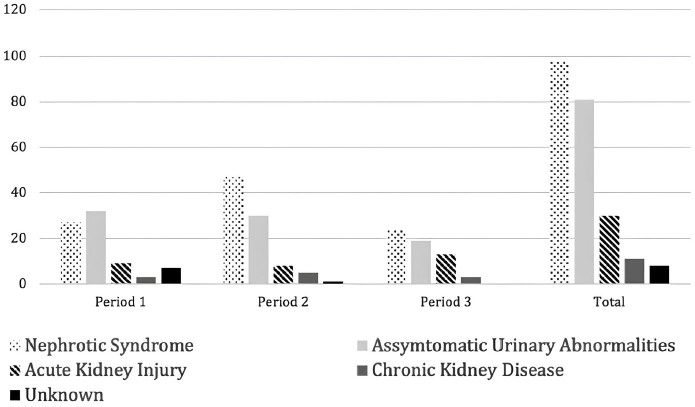
Indications for kidney biopsy. Numbers represent counts.

Indications for percutaneous KB and distribution over time are represented in Figure 2.

The most common indication for KB in the overall sample was NS in 42.9% of patients (n = 98), followed by asymptomatic urinary abnormalities in 35.5% (n = 81). Among patients with NS, 29 (29.5%) were classified as corticoresistant (CRNS) and 40 (40.8%) as corticodependent (CDNS). Acute kidney injury accounted for 13.2% (n = 30) of cases, while chronic kidney disease was a rare indication, justifying 4.8% (n = 11) of KB. As in the overall population, NS was the most common indication in time periods 2 and 3, as opposed to time period 1 when asymptomatic urinary abnormalities were more common.

A median of 15 (8–25) glomeruli were obtained. Immunofluorescence was performed in 89.3% of cases, increasing over time: 75.0% in period 1, 92.0% in period 2, and 100.0% in period 3 (p < 0.01). Electron microscopy was performed in 2.5% of cases.

**Figure 3:**
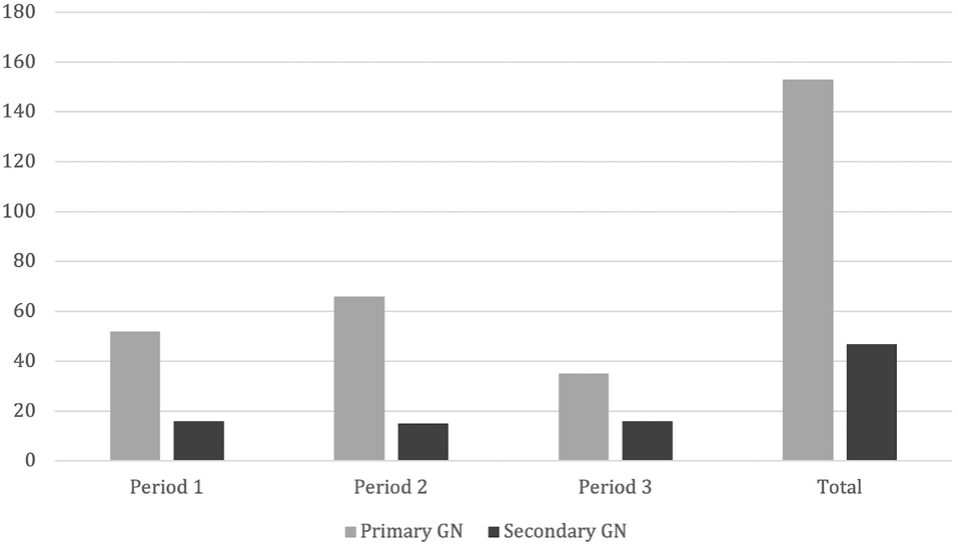
Distribution of primary and secondary GN. GN - Glomerulonephritis. Numbers represent counts.

Primary glomerular diseases were found in 67.1% of cases (n = 153) and were more frequent than secondary glomerular diseases in all time periods (Figure 3). Acute/chronic tubulointerstitial nephropathy was found in 3.5% (n = 8). There were seven cases of AS and one CNS.

**Figure 4:**
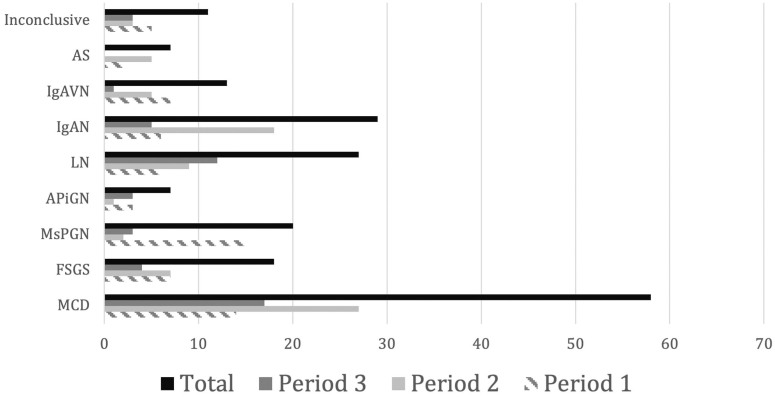
Evolution of the most common histological findings over the three time periods. MCD: Minimal Change Disease; IgAN: Immunoglobulin A Nephritis; LN: Lupus Nephritis; MsPGN: Mesangial Proliferative glomerulonephritis; FSGS: Focal Segmental Focal Glomerulosclerosis; IgAVN: Immunoglobulin A Vasculitis Nephritis; APiGN: Acute Post-infectious Glomerulonephritis; AS: Alport Syndrome. Numbers represent counts.

Table 2 represents the histologic diagnosis found and distribution over the three time periods and Figure 4 shows the relative distribution of the most common pathologies.

**Table 2:** Histological findings and evolution over the three time periods

	Period 1	Period 2	Period 3	Total
MCD	n = 14 (17.9%)	n = 27 (29.7%)	n = 17 (28.8%)	n = 58 (25.4%)
IgAN	n = 6 (7.7%)	n = 18 (19.8%)	n = 5 (8.5%)	n = 29 (12.7)
LN	n = 6 (7.7%)	n = 9 (9.9%)	n = 12 (20.3%)	n = 27 (11.8%)
MsPGN	n = 15 (19.2%)	n = 2 (2.2%)	n = 3 (5.1%)	n = 20 (8.8%)
FSGS	n = 7 (9.0%)	n = 7 (7.7%)	n = 4 (6.8%)	n = 18 (7.9%)
IgAVN	n = 7 (0.0%)	n = 5 (5.5%)	n = 1 (1.7%)	n = 13 (5.7%)
Inconclusive	n = 5 (6.4%)	n = 3 (3.3%)	n = 3 (5.1%)	n = 11 (4.8%)
CreGN	n = 5 (6.4%)	n = 1 (1.1%)	n = 2 (3.4%)	n = 8 (3.5%)
APiGN	n = 3 (3.8%)	n = 1 (1.1%)	n = 3 (5.1%)	n = 7 (3.1%)
AS	n = 2 (2.6%)	n = 5 (5.5%)	–	n = 7 (3.1%)
aTIN	n = 1 (1.3%)	n = 1 (1.1%)	n = 3 (5.1%)	n = 5 (2.2%)
IMN	n = 1 (1.3%)	n = 3 (3.3%)	n = 1 (1.7%)	n = 5 (2.2%)
MPGN	–	n = 4 (4.4%)	n = 1 (1.7%)	n = 5 (2.2%)
TBMD	n = 3 (3.8%)	n = 2 (2.2%)	–	n = 5 (2.2%)
cTIN	–	n = 1 (1.1%)	n = 2 (3.4%)	n = 3 (1.3%)
C3G	–	–	n = 2 (3.4)	n = 2 (0.9%)
IgMN	–	n = 2 (2.2%)	–	n = 2 (0.9%)
TM	n = 1 (1.3%)	–	–	n = 1 (0.4%)
C1qN	n = 1 (1.3%)	–	–	n = 1 (0.4%)
CNS	n = 1 (1.3%)	–	–	n = 1 (0.4%)
Total	n = 78	n = 91	n = 59	n = 228 (100%)

MCD: minimal change disease; IgAN: immunoglobulin A nephritis; LN: lupus nephritis; MsPGN: mesangial proliferative glomerulonephritis; FSGS: focal segmental glomerulosclerosis; IgAVN: immunoglobulin A vasculitis nephritis; CreGN: crescent glomerulonephritis; APiGN: acute post-infectious glomerulonephritis; AS: Alport syndrome; aTIN: acute tubulointerstitial nephritis; IMN: idiopathic membranous nephitis; MPGN: membranoproliferative glomerulonephritis; TBMD: thin basal membrane nephropathy; cTIN: chronic tubulointerstitial nephritis; C3G: C3 glomerulopathy; IgMN: immunoglobulin M nephropathy; TM: thrombotic microangiopathy; C1qN: C1q nephropathy; CNS: congenital nephrotic syndrome. Numbers represent count (percentages).

MCD was the most common pathology found (25.4%, n = 58), followed by IgAN (12.7%, n = 29), LN (11.8%, n = 27), MsPGN (8.8%, n = 20), FSGS (7.9%, n = 18), IgAVN (5.7%, n = 13), and creGN (3.5%, n = 8). APIGN and AS each accounted for 3.1% of cases. TBMD, MPGN, and IMN accounted for 2.2% each; C3N and IgMN for 0.9% each and C1qN, CNS, and TM 0.4% each. Tubulointerstitial nephritis was found in 8 patients (3.2%), 5 of which were acute (aTIN) and 3 were chronic (cTIN). We found that 4.8% of biopsies were inconclusive.

**Figure 5:**
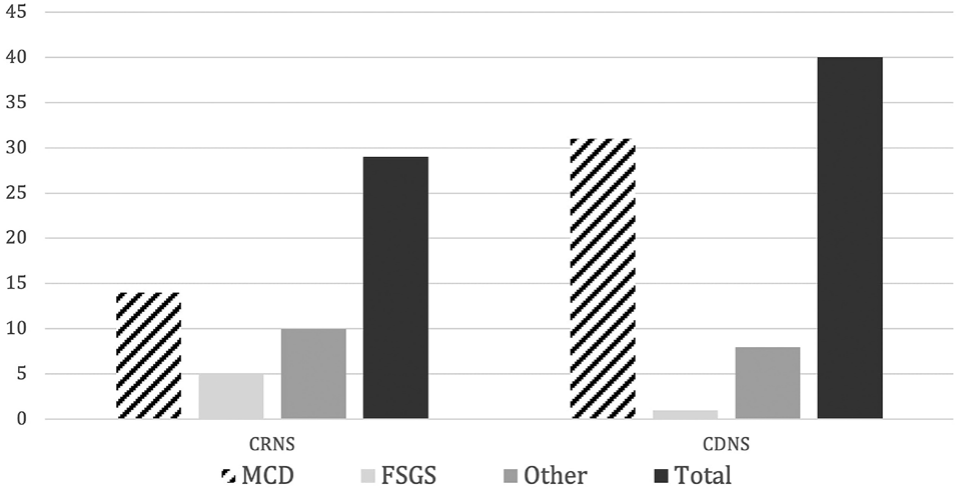
Distribution of most common pathologies in patients with corticoresistant and corticodependent nephrotic syndrome. MCD: Minimal Change Disease; FSGS: Focal Segmental Focal Glomerulosclerosis; CDNS: Corticodependent Nephrotic Syndrome; CRNS: Corticoresistant Nephrotic Syndrome. Numbers represent counts.

MCD was also the most common diagnosis in patients with NS, but it was found in 48.3% of patients with CRNS versus 77.5% of patients with CDNS, while FSGS was found in 2.5% of patients with CDNS versus 17.2% in patients with CRNS, although no statistical significant difference was found (p = 0.56) (Figure 5).

Among patients with asymptomatic urinary abnormalities, IgAN and LN were more frequently observed (24.7% and 22.2%, respectively).

MCD was the most common diagnosis in both sexes. Male to female ratio was 1:5.8 in LN, 10:1 in IgAN, 1:2.3 in IgAVN, 1.4:1 in MCD, 6:1 in APiGN, and 1.6:1 in FSGS.

Among children up to nine years old, the most common pathologies were MCD (n = 32, 33.7%), FSGS (n = 10, 10.5%), and MsPGN (n = 10, 10.5%). In adolescents (ten years old or older) MCD (n = 26, 20.2%), LN (n = 23, 17.8%), and IgAN (n = 18, 14.0%) were more commonly identified.

Over the three considered time periods, there were changes in diagnosis frequency. In period 1, the most common diagnoses were MsPGN, MCD, and IgAVN/FSGS; in period 2, MCD, IgAN, and LN; and in period 3 MCD, LN, and IgAN.

## Discussion

This was a retrospective study of all the first time percutaneous native kidney biopsies performed in our pediatric nephrology center in northern Portugal over the last 24 years, reporting the indications and pathological findings of 228 biopsies as well as the changes over this time period.

There was an increase in the number of biopsies from the first to the second period. This was likely due to the increased availability of ultrasound guiding. A decrease is seen from period 2 to period 3, which could be explained by stricter indications for KB and the rise in genetic testing. Despite the overall safety of KB when performed in patients without contraindications and by experienced teams, the procedure is not exempt from risks and should be reserved for patients in which empirical treatment is not the best option.

Median age at time of biopsy was in adolescence, which is consistent with similar studies and likely due to an important number of biopsies being performed on patients with NS presenting in atypical late age^9,11^. There were no differences in sex distribution, likely due to similar prevalence of MCD among sexes^9,12,13^. This is consistent with most studies, although a study in Serbia found a higher prevalence of females among patients submitted to pediatric percutaneous KB^8^ and some studies report a slight prevalence in males^9,14^.

As similarly reported in other studies, NS was the most important indication for KB in our sample^2,8,9,15,16^, and the second was asymptomatic urinary abnormalities. Reports from Italy, Israel, and England also mention proteinuria as the most common indication for biopsy^8,17^. In period 1, asymptomatic urinary abnormalities were the most common indication. This is no longer the case in most recent biopsies, when nephrotic syndrome became the most common indication. This was also found by Yin et al.^18^ and could be explained by increasing evidence that percutaneous KB is only indicated in persistent cases of gross hematuria^1^. In a study by Coppo et al.^9^, non-nephrotic proteinuria with hematuria was also a more common indication for KB than nephrotic range proteinuria. AKI and CKD are rarer indications for KB, as reported in other studies^9,11^.

The median of obtained glomeruli was sufficient for diagnosis and increased over time, suggesting satisfactory technical quality. Immunofluorescence use increased over time, reaching 100% in period 3, due to its increasing recognized importance in diagnosis.

Electron microscopy was reserved for selected cases, because of its additional cost and unavailability in earlier years.

Primary glomerular diseases were more frequent than secondary GN, as reported in several studies^2,9,18^, mostly due to the high prevalence of MCD. LN was the most significant contributor in secondary GN. Acute/chronic tubulointerstitial nephropathy are rarer diagnoses in the pediatric population, acute cases being more frequent than chronic.

In our study, MCD was the most common pathology (25.4%). This is in accordance with multiple studies reporting MCD as the most common GN in children^12,15,19,20,21,22^. Previous reports in Portugal suggest IgA vasculitis is the most common primary GN in adults^23^.

However, histopathological prevalence varies greatly according to geographic location and some reports point IgAN^6,9,11^ and IgAVN^24^ as the most common diagnosis. Moreover, FSGS is the most common pathology in reports from Turkey, Greece, Pakistan, and Serbia, which could be related to stricter indications for KB^10,25,26^. Demircin et al.^2^ reports MPGN as the most common pathology.

We found a higher-than-expected prevalence of MCD among patients with CRNS. Although more frequent in adults, IgAN was the second most common pathology identified (12.7%, n = 29), which is less common than reported by Coppo et al.^9^ (18.8%). Despite MCD being the most common diagnosis in both sexes, LN was more common in females than males, as expected. The prevalence of LN (11.8%) was higher than that of reports in China and Italy (5%) and lower than reported by Yuen et al.^13^ (23%)^9,18^. This could be related to differences in ultraviolet radiation exposure associated with different geographic locations and cultural differences^26^. MsPGN prevalence was higher than reported by other studies (3–5%)^12,13^. FSGS incidence was similar to other reports (7.9%)^2,9,13^. IgAVN was significantly less common (5.7%) than in several other reports^2,9,18^. APiGN, CreGN, and AS prevalence were similar to previous reports^2,9,15^. TBMD prevalence was lower than found by Coppo et al.^9^ and Yuen et al.^13^. MPGN incidence was much lower than in different reports^2,12,26^. IMN prevalence was also lower in our sample (2.2%)^15^. IgMN and C1q nephropathy were found less frequently than previously reported^2,13^. C3N, congenital NS, and thrombotic microangiopathy were found in similar proportion to previous reports^2,15,27^.

In patients with NS, MCD was also the most common diagnosis, as opposed to studies which found FSGS to be the most common diagnosis in CRNS and no cases of MCD in this group^8,14,28,29^. However, FSGS was more common in those with CRNS than those with CDNS in our sample.

In our study, FSGS accounted for 7.9% of cases, which is higher than that reported by Yin et al.^18^ in 2013, but lower than reported by Fidan et al.^26^ and Printza et al.^16^. Some studies report an increase in FSGS in recent years, possibly due to increasing rates of obesity among the general population as well as in children^14^. However, we did not find an increase in FSGS prevalence overtime.

We found that 4.8% of biopsies were inconclusive. Insufficient glomeruli in the sample and the lack of electron microscopy likely contributed to these results. Also, samples in which FSGS was identified could be associated with other pathologies. Genetic testing has had an increasing role in assisting diagnosis in such cases. In the future, the role of genetic testing in etiology investigation is likely to be more predominant.

MCD was the most common diagnosis in both sexes. As reported by other studies, LN was most common in females, with a 1:5.8 ratio^18^. The difference is milder than in adults possibly because of lower estrogen levels in female children^18^.

Most common diagnoses varied over time: MsPGN, MCD and IgAVN/FSGS in period 1 and MCD, LN, and IgAN in period 3.

Our study is not without limitations. Despite the important number of biopsies, the single center design provides limited information on pediatric renal pathology in Portugal. A national study would be of interest to better understand relative frequencies of different pathologies in our population. The difference in the number of biopsies among groups could influence pathology prevalence. The retrospective nature compromised the recollection of partial clinical data.

In conclusion, in this pediatric population studied in Northern Portugal, NS was the most common indication for KB and MCD was the most frequently found pathology.
