# Supplementary material for: Percutaneous kidney biopsies in children: a 24-year review in a tertiary center in northern Portugal
Source: J Bras Nefrol. 2024 Apr 8;46(3):e20230143. doi: 10.1590/2175-8239-JBN-2023-0143en (PMC11287898; doi:10.1590/2175-8239-JBN-2023-0143en)
Supplement: Translation

# Translation: Biópsias renais percutâneas em crianças: uma revisão de 24 anos em um centro terciário no norte de Portugal

**DOI:** 10.1590/2175-8239-JBN-2023-0143pt

**Authors:** Patrícia Sousa, Catarina Brás, Catarina Menezes, Ramon Vizcaino, Teresa Costa, Maria Sameiro Faria, Conceição Mota

**Affiliations:** 1 Hospital Senhora da Oliveira, Serviço de Pediatria, Guimarães, Portugal.; 2 Hospital Professor Doutor Fernando Fonseca, Serviço de Nefrologia, Lisboa, Portugal.; 3 Centro Hospitalar Universitário de Santo António, Centro Materno-Infantil do Norte, Serviço de Pediatria, Porto, Portugal.; 4 Centro Hospitalar Universitário de Santo António, Serviço de Anatomia Patológica, Porto, Portugal.; 5 Centro Hospitalar Universitário de Santo António, Centro Materno-Infantil do Norte, Unidade de Nefrologia Pediátrica, Serviço de Pediatria, Porto, Portugal.; 6 Unidade de Ciências Biomoleculares Aplicadas, Department of Biological Science, Porto, Portugal.

**Keywords:** Biópsia; Rim, Pediatria

## Abstract

**Introdução:**

A biópsia renal (BR) percutânea é fundamental para diagnóstico e manejo de diversas patologias renais. Dados nacionais sobre BR nativa em pacientes pediátricos são escassos. Nosso objetivo foi revisar características demográficas, clínicas e padrões histopatológicos em crianças submetidas a BR percutânea nativa ao longo de 24 anos.

**Métodos:**

Estudo observacional retrospectivo de pacientes submetidos a BR percutâneas nativas em unidade de nefrologia pediátrica entre 1998 e 2021, comparando três períodos: período 1 (1998–2005), período 2 (2006–2013), período 3 (2014–2021).

**Resultados:**

Constatamos que foram realizadas 228 BR, 78 (34,2%) no período 1, 91 (39,9%) no período 2, 59 (25,9%) no período 3. A idade mediana na BR foi 11 (7–14) anos. As principais indicações para BR foram síndrome nefrótica (SN) (42,9%), hematúria e/ou proteinúria não nefrótica (35,5%), lesão renal aguda (13,2%). Glomerulopatias primárias foram mais frequentes (67,1%), principalmente doença de lesão mínima (DLM) (25,4%), nefropatia por IgA (12,7%), glomerulonefrite mesangioproliferativa (GN) (8,8%). Das glomerulopatias secundárias, nefrite lúpica (NL) foi a mais prevalente (11,8%). No grupo 1, hematúria e/ou a proteinúria não nefrótica foram os principais motivos para BR, ao contrário da SN nos grupos 2 e 3 (p < 0,01). A NL apresentou tendência crescente (período 1–3: 2,6%–5,3%) e a glomeruloesclerose segmentar focal (GESF) apresentou leve tendência decrescente (período 1–3: 3,1%–1,8%), sem significância estatística.

**Conclusões:**

A principal indicação para BR foi SN, que aumentou ao longo do tempo, justificando o achado de DLM como principal diagnóstico histológico. A NL apresentou aumento na incidência ao longo do tempo, enquanto os casos de GESF não aumentaram.

## Introdução

A biópsia renal (BR) percutânea desempenha um papel importante no diagnóstico de doenças renais, fornecendo dados histopatológicos para complementar a avaliação clínica e auxiliar no diagnóstico, tratamento e prognóstico adequados^1^. A biópsia renal tem se mostrado um procedimento seguro, uma vez que são raras as complicações graves após o procedimento, sendo as mais comuns o sangramento: hematoma perirrenal (12,4%) e hematúria macroscópica (2,6%)^2–4^. A hematúria microscópica é encontrada em até 3,5% dos pacientes. Menos de 1% dos pacientes necessitam de transfusão de hemácias^5^.

Diversos estudos mostram diferenças na epidemiologia da doença renal entre populações adultas e pediátricas, bem como variações geográficas^2,6^. Os dados referentes à população em geral estão amplamente disponíveis, enquanto os registros pediátricos internacionais e nacionais são mais recentes e escassos^7^. Portanto, relatos sobre a epidemiologia e histopatologia da doença renal crônica em crianças são fundamentais para orientar nossa abordagem.

A glomerulonefrite representa um fardo significativo na doença renal crônica pediátrica, perdendo apenas para as anomalias congênitas do rim e do trato urinário^1^.

Estudos realizados em diferentes populações apontaram a doença de lesão mínima (DLM), a nefrite por imunoglobulina A (NIgA) e a nefrite-vasculite por imunoglobulina A (NVIgA) como os achados histopatológicos mais comuns. A indicação mais frequente para BR em crianças parece ser a síndrome nefrótica (SN)^1^ e/ou a proteinúria^8^, embora alguns estudos relatem hematúria como a mais comum^9^.

Neste estudo, nosso objetivo foi relatar as indicações clínicas e os achados histopatológicos da BR percutânea nativa em crianças em um centro pediátrico terciário no norte de Portugal, bem como analisar a evolução ao longo de um período de vinte e quatro anos.

## Métodos

Este estudo retrospectivo incluiu todos os pacientes submetidos a uma primeira BR percutânea nativa em um centro terciário de nefrologia pediátrica ao norte de Portugal, de 1º de janeiro de 1998 a 31 de dezembro de 2021. Foram excluídas biópsias repetidas no mesmo paciente e biópsias realizadas em rins transplantados. Todas as BR foram realizadas com orientação por ultrassom, por nefrologistas pediátricos, até 2012 e por radiologistas intervencionistas a partir de então, devido à reforma organizacional. Não houve outras alterações significativas no método de biópsia durante esse período. O consentimento informado por escrito foi obtido para todos os procedimentos.

Os registros clínicos digitais e impressos dos pacientes incluídos foram revisados para recuperar dados referentes a sexo, idade no momento do diagnóstico, idade no momento da biópsia, indicação de BR percutânea, presença e grau de hematúria, presença e grau de proteinúria, número de glomérulos obtidos, coloração por imunofluorescência, microscopia eletrônica e diagnóstico histológico. As indicações para BR percutânea foram categorizadas como SN, anomalias urinárias assintomáticas (incluindo proteinúria não nefrótica, hematúria ou ambas), insuficiência renal aguda (IRA) e insuficiência renal crônica (DRC).

As amostras foram examinadas por patologistas experientes e foram consideradas válidas se houvesse pelo menos 7 glomérulos ou, caso contrário, se um diagnóstico fosse feito de acordo com o protocolo institucional e a literatura^10^.

Foi realizada uma coloração por imunofluorescência usando antissoros policlonais contra IgG, IgM, IgA, C3, C4 e C1q e albumina humana.

As doenças renais foram divididas em quatro grupos: 1) glomerulonefrite primária (GN); 2) GN secundária; 3) doenças tubulointersticiais e 4) outras doenças.

A GN primária incluiu: glomerulonefrite crescêntica (GNC), nefropatia por C1q (NC1q), glomerulopatia C3 (GC3), glomeruloesclerose segmentar focal (GESF), nefropatia membranosa idiopática (NMi), NIgA, nefropatia por imunoglobulina M (NIgM), glomerulonefrite membranoproliferativa (GNMP), glomerulonefrite mesangioproliferativa (GNMsP), DLM e nefropatia da membrana basal fina (NMBF). A GN secundária incluiu glomerulonefrite pós-infecciosa aguda (GNPIA), NVIgA e nefrite lúpica (NL). As doenças tubulointersticiais incluíram nefrite tubulointersticial aguda e crônica. Outros diagnósticos incluem microangiopatia trombótica (MT), síndrome de Alport (SA) e síndrome nefrótica congênita (SNC).

A amostra foi dividida em três períodos de tempo, de acordo com o momento da biópsia: período 1 (1998–2005), período 2 (2006–2013) e período 3 (2014–2021).

Para variáveis categóricas, os dados são apresentados como frequências e porcentagens. As variáveis contínuas apresentaram uma distribuição não paramétrica e são reportadas como medianas e intervalos interquartis. Os grupos foram comparados de acordo com o teste U de Mann-Whitney. Um valor de *p* < 0,05 foi considerado estatisticamente significativo. A análise estatística foi realizada utilizando o software IBM SPSS *Statistics* versão 28.0.1.0 (SPSS Inc., Chicago, IL, EUA).

O estudo foi aprovado pelo Comitê de Ética da instituição e o consentimento individual informado por escrito foi considerado desnecessário para este estudo retrospectivo.

## Resultados

No total, foram realizadas 228 BR nativas, 78 (34,2%%) no período 1, 91 (39,9%) no período 2 e 59 (25,9%) no período 3. A distribuição por ano está representada na Figura 1.

**Figura 1:** Evolução das biópsias renais percutâneas realizadas de 1998 a 2021. Os pontos representam a contagem de biópsias por ano.

As características demográficas estão representadas na Tabela 1. Em nossa amostra, 50,4% dos pacientes eram do sexo masculino (n = 115) e 49,6% do sexo feminino (n = 113). A distribuição por sexo foi semelhante nos três períodos de tempo. A mediana de idade no momento da biópsia foi de 11 anos (7–14), sem diferença significativa entre os períodos.

**Tabela 1:** Características demográficas

		Período 1	Período 2	Período 3	Total
Espécimes		n = 78 (34.2%)	n = 91 (39.9%)	n = 59 (25.9%)	n = 228
Sexo	Masculino	n = 37 (47.4%)	n = 49 (53.8%)	n = 29 (49.2%)	n = 115 (50.4%)
	Feminino	n = 41 (52.6%)	n = 42 (46.2%)	n = 30 (50.8%)	n = 113 (49.6%)
Age (years)		9 (6–13)	12 (8–14)	11 (7–14)	11 (7–14)

Os números representam a contagem (porcentagens).

As indicações para BR percutânea e a distribuição ao longo do tempo estão representadas na Figura 2.

**Figura 2:** Indicações para biópsia renal. Os números representam as contagens.

A indicação mais comum para BR na amostra geral foi a SN em 42,9% dos pacientes (n = 98), seguida por anomalias urinárias assintomáticas em 35,5% (n = 81). Entre os pacientes com SN, 29 (29,5%) foram classificados como corticorresistentes (SNCR) e 40 (40,8%) como corticodependentes (SNCD). A lesão renal aguda foi responsável por 13,2% (n = 30) dos casos, enquanto a doença renal crônica foi uma indicação rara, justificando 4,8% (n = 11) das BR. Da mesma forma que na população em geral, a SN foi a indicação mais comum nos períodos 2 e 3, ao contrário do período 1, quando as anomalias urinárias assintomáticas foram mais comuns.

Obteve-se uma mediana de 15 (8–25) glomérulos. A imunofluorescência foi realizada em 89,3% dos casos, aumentando ao longo do tempo: 75,0% no período 1, 92,0% no período 2 e 100,0% no período 3 (p < 0,01). A microscopia eletrônica foi realizada em 2,5% dos casos.

As doenças glomerulares primárias foram detectadas em 67,1% dos casos (n = 153) e foram mais frequentes do que as doenças glomerulares secundárias em todos os períodos (Figura 3). A nefropatia tubulointersticial aguda/crônica foi encontrada em 3,5% (n = 8). Houve sete casos de SA e um de SNC.

**Figura 3:** Distribuição de GN primária e secundária. GN - Glomerulonefrite. Os números representam as contagens.

A Tabela 2 representa o diagnóstico histológico encontrado e a distribuição ao longo dos três períodos de tempo e a Figura 4 mostra a distribuição relativa das patologias mais comuns.

**Tabela 2:** Achados histológicos e evolução ao longo dos três períodos de tempo

	Período 1	Período 2	Período 3	Total
MCD	n = 14 (17.9%)	n = 27 (29.7%)	n = 17 (28.8%)	n = 58 (25.4%)
IgAN	n = 6 (7.7%)	n = 18 (19.8%)	n = 5 (8.5%)	n = 29 (12.7)
LN	n = 6 (7.7%)	n = 9 (9.9%)	n = 12 (20.3%)	n = 27 (11.8%)
MsPGN	n = 15 (19.2%)	n = 2 (2.2%)	n = 3 (5.1%)	n = 20 (8.8%)
FSGS	n = 7 (9.0%)	n = 7 (7.7%)	n = 4 (6.8%)	n = 18 (7.9%)
IgAVN	n = 7 (0.0%)	n = 5 (5.5%)	n = 1 (1.7%)	n = 13 (5.7%)
Inconclusive	n = 5 (6.4%)	n = 3 (3.3%)	n = 3 (5.1%)	n = 11 (4.8%)
CreGN	n = 5 (6.4%)	n = 1 (1.1%)	n = 2 (3.4%)	n = 8 (3.5%)
APiGN	n = 3 (3.8%)	n = 1 (1.1%)	n = 3 (5.1%)	n = 7 (3.1%)
AS	n = 2 (2.6%)	n = 5 (5.5%)	–	n = 7 (3.1%)
aTIN	n = 1 (1.3%)	n = 1 (1.1%)	n = 3 (5.1%)	n = 5 (2.2%)
IMN	n = 1 (1.3%)	n = 3 (3.3%)	n = 1 (1.7%)	n = 5 (2.2%)
MPGN	–	n = 4 (4.4%)	n = 1 (1.7%)	n = 5 (2.2%)
TBMD	n = 3 (3.8%)	n = 2 (2.2%)	–	n = 5 (2.2%)
cTIN	–	n = 1 (1.1%)	n = 2 (3.4%)	n = 3 (1.3%)
C3G	–	–	n = 2 (3.4)	n = 2 (0.9%)
IgMN	–	n = 2 (2.2%)	–	n = 2 (0.9%)
TM	n = 1 (1.3%)	–	–	n = 1 (0.4%)
C1qN	n = 1 (1.3%)	–	–	n = 1 (0.4%)
CNS	n = 1 (1.3%)	–	–	n = 1 (0.4%)
Total	n = 78	n = 91	n = 59	n = 228 (100%)

DLM: doença de lesão mínima; NIgA: nefrite por imunoglobulina A; NL: nefrite lúpica; GNMsP: glomerulonefrite mesangioproliferativa; GESF: glomeruloesclerose segmentar focal; NVIgA: nefrite-vasculite por imunoglobulina A; GNC: glomerulonefrite crescêntica; GNPIA: glomerulonefrite pós-infecciosa aguda; SA: síndrome de Alport; NTIa: nefrite tubulointersticial aguda; NMi: nefropatia membranosa idiopática; GNMP: glomerulonefrite membranoproliferativa; NMBF: nefropatia da membrana basal fina; NTIc: nefrite tubulointersticial crônica; GC3: glomerulopatia C3; NIgM: nefropatia por imunoglobulina M; MT: microangiopatia trombótica; NC1q: nefropatia por C1q; SNC: síndrome nefrótica congênita. Os números representam a contagem (porcentagens).

**Figura 4:** Evolução dos achados histológicos mais comuns nos três períodos de tempo. DLM: Doença de Lesão Mínima; NIgA: Nefrite por Imunoglobulina A; NL: Nefrite Lúpica; GNMsP: Glomerulonefrite Mesangioproliferativa; GESF: Glomeruloesclerose Segmentar Focal; NVIgA: Nefrite-Vasculite por Imunoglobulina A; GNPIA: Glomerulonefrite Pós-infecciosa Aguda; SA: Síndrome de Alport. Os números representam as contagens.

A DLM foi a patologia mais comum encontrada (25,4%; n = 58), seguida por NIgA (12,7%; n = 29), NL (11,8%; n = 27), GNMsP (8,8%; n = 20), GESF (7,9%; n = 18), NVIgA (5,7%; n = 13) e GNC (3,5%; n = 8). A GNPIA e a SA representaram, cada uma, 3,1% dos casos. NMBF, GNMP e NMi representaram 2,2% cada; NC3 e NIgM, 0,9% cada e NC1q, SNC e MT, 0,4% cada. A nefrite tubulointersticial foi encontrada em 8 pacientes (3,2%), sendo 5 agudas (NTIa) e 3 crônicas (NTIc). Constatamos que 4,8% das biópsias foram inconclusivas.

A DLM também foi o diagnóstico mais comum em pacientes com SN, mas foi encontrada em 48,3% dos pacientes com SNCR *versus* 77,5% dos pacientes com SNCD, enquanto a GESF foi encontrada em 2,5% dos pacientes com SNCD *versus* 17,2% em pacientes com SNCR, embora não se tenha encontrado diferença estatisticamente significativa (p = 0,56) (Figura 5).

**Figura 5:** Distribuição das patologias mais comuns em pacientes com síndrome nefrótica corticorresistente e corticodependente. DLM: Doença de Lesão Mínima; GESF: Glomeruloesclerose Segmentar Focal; SNCD: Síndrome Nefrótica Corticodependente; SNCR: Síndrome Nefrótica Corticorresistente. Os números representam as contagens.

Entre os pacientes com anomalias urinárias assintomáticas, a NIgA e a NL foram observadas com maior frequência (24,7% e 22,2%, respectivamente).

A DLM foi o diagnóstico mais comum em ambos os sexos. A proporção entre homens e mulheres foi de 1:5,8 em NL, 10:1 em NIgA, 1:2,3 em NVIgA, 1,4:1 em DLM, 6:1 em GNPIA e 1,6:1 em GESF.

Entre crianças de até nove anos de idade, as patologias mais comuns foram DLM (n = 32; 33,7%), GESF (n = 10; 10,5%) e GNMsP (n = 10; 10,5%). Em adolescentes (dez anos de idade ou mais), a DLM (n = 26; 20,2%), a NL (n = 23; 17,8%) e a NIgA (n = 18; 14,0%) foram mais comumente identificadas.

Ao longo dos três períodos de tempo considerados, houve alterações na frequência do diagnóstico. No período 1, os diagnósticos mais comuns foram GNMsP, DLM e NVIgA/GESF; no período 2, DLM, NIgA e NL; e no período 3, DLM, NL e NIgA.

## Discussão

Este foi um estudo retrospectivo de todas as biópsias percutâneas de rim nativo realizadas pela primeira vez em nosso centro de nefrologia pediátrica no norte de Portugal nos últimos 24 anos, relatando as indicações e os achados patológicos de 228 biópsias, bem como as alterações ao longo desse período.

Houve um aumento no número de biópsias do primeiro para o segundo período. Isso foi provavelmente devido à maior disponibilidade de orientação por ultrassom. Observa-se uma redução do período 2 para o período 3, que pode ser explicada por indicações mais rigorosas para BR e pelo aumento dos testes genéticos. Apesar da total segurança da BR quando realizada em pacientes sem contraindicações e por equipes experientes, o procedimento não está isento de riscos e deve ser reservado para pacientes nos quais o tratamento empírico não é a melhor opção.

A idade mediana no momento da biópsia foi a adolescência, o que é consistente com estudos semelhantes e provavelmente devido a um número importante de biópsias realizadas em pacientes com SN que se apresentam em idade tardia atípica^9,11^. Não houve diferenças na distribuição por sexo, provavelmente devido à prevalência semelhante de DLM entre os sexos^9,12,13^. Isso está em consonância com a maioria dos estudos, embora um estudo na Sérvia tenha encontrado uma prevalência maior de mulheres entre os pacientes submetidos à BR^8^ percutânea pediátrica, e alguns estudos relatem uma ligeira prevalência de homens^9,14^.

Como relatado de forma semelhante em outros estudos, a SN foi a indicação mais importante para BR em nossa amostra^2,8,9,15,16^, sendo a segunda as anomalias urinárias assintomáticas. Relatos da Itália, Israel e Inglaterra também mencionam a proteinúria como a indicação mais comum para biópsia^8,17^. No período 1, as anomalias urinárias assintomáticas foram a indicação mais comum. Esse não é o caso em biópsias mais recentes, quando a síndrome nefrótica se tornou a indicação mais comum. Essa constatação também foi feita por Yin et al.^18^ e pode ser explicada pela crescente evidência de que a BR percutânea é somente indicada em casos persistentes de hematúria macroscópica^1^. Em um estudo realizado por Coppo et al.^9^, a proteinúria não nefrótica com hematúria também foi uma indicação mais comum para BR do que a proteinúria na faixa nefrótica. A LRA e a DRC são indicações mais raras para BR, conforme relatado em outros estudos^9,11^.

A mediana dos glomérulos obtidos foi suficiente para o diagnóstico e aumentou ao longo do tempo, sugerindo qualidade técnica satisfatória. O uso da imunofluorescência aumentou com o passar do tempo, atingindo 100% no período 3, devido à sua importância cada vez mais reconhecida no diagnóstico.

A microscopia eletrônica foi reservada para casos selecionados, devido ao seu custo adicional e à indisponibilidade em anos anteriores.

As doenças glomerulares primárias foram mais frequentes do que a GN secundária, conforme relatado em diversos estudos^2,9,18^, principalmente devido à alta prevalência de DLM. A NL foi o fator de contribuição mais significativo na GN secundária. A nefropatia tubulointersticial aguda/crônica são diagnósticos mais raros na população pediátrica, sendo os casos agudos mais frequentes que os crônicos.

Em nosso estudo, a DLM foi a patologia mais comum (25,4%). Isso está de acordo com diversos estudos que relatam a DLM como a GN mais comum em crianças^12,15,19–22^. Registros anteriores em Portugal sugerem que a vasculite por IgA é a GN primária mais comum em adultos^23^.

No entanto, a prevalência histopatológica varia muito de acordo com a localização geográfica e alguns relatos apontam a NIgA^6,9,11^ e a NVIgA^24^ como o diagnóstico mais comum. Além disso, a GESF é a patologia mais comum em relatos da Turquia, Grécia, Paquistão e Sérvia, o que pode estar relacionado a indicações mais rigorosas para BR^10,25,26^. Demircin et al.^2^ relatam a GNMP como a patologia mais comum.

Encontramos uma prevalência maior do que a esperada de DLM entre os pacientes com SNCR. Embora mais frequente em adultos, a NIgA foi a segunda patologia mais comum identificada (12,7%; n = 29), sendo menos comum do que a relatada por Coppo et al.^9^ (18,8%). Apesar de a DLM ser o diagnóstico mais comum em ambos os sexos, a NL foi mais comum em mulheres do que em homens, como esperado. A prevalência de NL (11,8%) foi maior do que a relatada na China e na Itália (5%) e inferior à relatada por Yuen et al.^13^ (23%)^9,18^. Isso pode estar relacionado a diferenças na exposição à radiação ultravioleta associada a diferentes localizações geográficas e diferenças culturais^26^. A prevalência de GNMsP foi maior do que a relatada por outros estudos (3–5%)^12,13^. A incidência de GESF foi semelhante à de outros relatos (7,9%)^2,9,13^. A NVIgA foi significativamente menos comum (5,7%) do que em diversos outros relatos^2,9,18^. A prevalência de GNPIA, GNC e SA foi semelhante à de relatos anteriores^2,9,15^. A prevalência de NMBF foi menor do que a encontrada por Coppo et al.^9^ e Yuen et al.^13^. A incidência de GNMP foi muito menor do que em diferentes relatos^2,12,26^. A prevalência de NMi também foi menor em nossa amostra (2,2%)^15^. A NIgM e a nefropatia por C1q foram encontradas com menos frequência do que o relatado anteriormente^2,13^. NC3, SN congênita e microangiopatia trombótica foram encontradas em proporção semelhante aos relatos anteriores^2,15,27^.

Em pacientes com SN, a DLM também foi o diagnóstico mais comum, ao contrário de estudos que constataram que a GESF foi o diagnóstico mais comum na SNCR e nenhum caso de DLM nesse grupo^8,14,28,29^. No entanto, em nossa amostra, a GESF foi mais comum em pacientes com SNCR do que naqueles com SNCD.

Em nosso estudo, a GESF foi responsável por 7,9% dos casos, valor superior ao relatado por Yin et al.^18^ em 2013, mas inferior ao relatado por Fidan et al.^26^ e Printza et al.^16^. Alguns estudos relatam um aumento na GESF nos últimos anos, possivelmente devido ao aumento das taxas de obesidade na população em geral, bem como em crianças^14^. No entanto, não encontramos um aumento na prevalência de GESF ao longo do tempo.

Constatamos que 4,8% das biópsias foram inconclusivas. O número insuficiente de glomérulos na amostra e a falta de microscopia eletrônica provavelmente contribuíram para esses resultados. Além disso, as amostras nas quais a GESF foi identificada podem estar associadas a outras patologias. Os testes genéticos têm desempenhado um papel crescente no auxílio ao diagnóstico nesses casos. No futuro, é provável que o papel dos testes genéticos na investigação da etiologia seja mais predominante.

A DLM foi o diagnóstico mais comum em ambos os sexos. Conforme relatado por outros estudos, a NL foi mais comum em mulheres, com uma proporção de 1:5,8^18^. A diferença é mais branda do que em adultos, possivelmente devido aos níveis mais baixos de estrogênio em crianças do sexo feminino^18^.

Os diagnósticos mais frequentes variaram ao longo do tempo, destacando-se GNMsP, DLM e NVIgA/GESF no período 1 e DLM, NL e NIgA no período 3.

Nosso estudo não está isento de limitações. Apesar do importante número de biópsias, o desenho de centro único fornece informações limitadas sobre a patologia renal pediátrica em Portugal. Um estudo nacional seria de interesse para uma melhor compreensão das frequências relativas de diferentes patologias em nossa população. A diferença no número de biópsias entre os grupos pode influenciar a prevalência da patologia. A natureza retrospectiva comprometeu a coleta de dados clínicos parciais.

Concluindo, nessa população pediátrica estudada no norte de Portugal, a SN foi a indicação mais comum para BR e a DLM foi a patologia mais frequentemente encontrada.
